# Brain explorer for connectomic analysis

**DOI:** 10.1007/s40708-017-0071-9

**Published:** 2017-08-23

**Authors:** Huang Li, Shiaofen Fang, Joey A. Contreras, John D. West, Shannon L. Risacher, Yang Wang, Olaf Sporns, Andrew J. Saykin, Joaquín Goñi, Li Shen, for the Alzheimer’s Disease Neuroimaging Initiative

**Affiliations:** 1 Department of Radiology and Imaging Sciences, Indiana University School of Medicine, Indianapolis, IN USA; 2 Department of Computer and Information Science, Indiana University-Purdue University Indianapolis, Indianapolis, IN USA; 3 Department of Radiology Imaging Research, Medical College of Wisconsin, Milwaukee, WI USA; 4 Department of Psychological and Brain Sciences, Indiana University Bloomington, Bloomington, IN USA; 5 School of Industrial Engineering, Purdue University, West Lafayette, IN USA; 6 Weldon School of Biomedical Engineering, Purdue University, West Lafayette, IN USA; 7 Purdue Institute for Integrative Neuroscience, Purdue University, West Lafayette, IN USA

**Keywords:** Brain connectome, Magnetic resonance imaging, Diffusion tensor imaging, Functional magnetic resonance imaging, Visualization

## Abstract

Visualization plays a vital role in the analysis of multimodal neuroimaging data. A major challenge in neuroimaging visualization is how to integrate structural, functional, and connectivity data to form a comprehensive visual context for data exploration, quality control, and hypothesis discovery. We develop a new integrated visualization solution for brain imaging data by combining scientific and information visualization techniques within the context of the same anatomical structure. In this paper, new surface texture techniques are developed to map non-spatial attributes onto both 3D brain surfaces and a planar volume map which is generated by the proposed volume rendering technique, spherical volume rendering. Two types of non-spatial information are represented: (1) time series data from resting-state functional MRI measuring brain activation; (2) network properties derived from structural connectivity data for different groups of subjects, which may help guide the detection of differentiation features. Through visual exploration, this integrated solution can help identify brain regions with highly correlated functional activations as well as their activation patterns. Visual detection of differentiation features can also potentially discover image-based phenotypic biomarkers for brain diseases.

## Introduction

Human connectomics [1] is an emerging field that holds great promise for a systematic characterization of human brain connectivity and its relationship with cognition and behavior. The analysis of human brain connectome networks faces two major challenges: (1) how to reliably and accurately identify connectivity patterns related to cognition, behavior, and also neurological conditions based on an unknown set of network characterization and features; (2) how to seamlessly integrate computational methods with human knowledge and how to translate this into user-friendly, interactive software tools that optimally combines human expertise and machine intelligence to enable novel contextually meaningful discoveries. Both challenges require the development of highly interactive and comprehensive visualization tools that can guide researchers through a complex sea of data and information for knowledge discovery.

Scientific visualization has traditionally been playing a role of visually interpreting and displaying complex scientific data, such as medical image data, to reveal structural and material details so as to help the understanding of the scientific phenomena. Example studies include diffusion tensor imaging (DTI) fiber tract visualization [2–7], network visualization [8–11], and multimodal data visualization [12–14]. In this context, recent development in information visualization provides new ways to visualize non-structural attributes or in-depth analysis data, such as graph/network visualization and time series data visualization. These, however, are usually separate visual representations away from the anatomical structures, which are limited at providing effective support for visual exploration of multimodal brain data.

To remedy the visual inefficiency and maximize human cognitive abilities during visual exploration, this paper proposes to integrate the visual representations of the connectome network attributes onto the surfaces of the anatomical structures of human brain. Multiple visual encoding schemes, combined with various interactive visualization tools, can provide an effective and dynamic data exploration environment for neuroscientists to better identify patterns, trends and markers. In addition, we develop a spherical volume rendering (SVR) algorithm using omni-directional ray casting and information-encoded texture mapping. It provides a single 2D map of the entire rendered volume to provide better support for global visual evaluation and feature selection for analysis purpose.

Our primary contributions in this work include:

1. Development of a method to represent rich attribute information using information-encoded textures.
2. Development of a new spherical volume rendering (SVR) technique that can generate a complete and camera-invariant view (volume map) of the entire structure.
3. Application of this approach to human brain visualization. Our experiments show great potential that this approach can be very useful in the analysis of neuroimaging data.

In the rest of this paper, we first discuss previous work related to this topic in Sect. 2. In Sect. 3, we will describe the data we used in this study. In Sect. 4, we will present technical details of encoded textures to visualize rich attribute information. In Sect. 5, we will present technical details and results of the SVR algorithm. Some implementation details and visualization evaluation will be provided in Sect. 6. We conclude the paper in Sect. 7 with our final remarks and future work.

## Related work

Human brain connectomics involves several different imaging modalities that require different visualization techniques. More importantly, multimodal visualization techniques need to be developed to combine the multiple modalities and present both details and context for connectome-related data analysis. Margulies et al. [3] provided an excellent overview of the various available visualization tools for brain anatomical and functional connectivity data. Some of these techniques are capable of carrying out multimodal visualization involving magnetic resonance imaging (MRI), fiber tracts as obtained from DTI and overlaying network connections. Various graphics rendering tools, along with special techniques such as edge bundling (to reduce clutter), have been applied to visualize DTI fiber tracts [2–5]. Due to tracking uncertainties in DTI fibers, these deterministic rendering can sometimes be misleading. Hence, rendering techniques for probabilistic DTI tractography have also been proposed [6, 7]. Several techniques have been developed to provide anatomical context around the DTI fiber tracts [12–14]. This typically requires semitransparent rendering with carefully defined transfer functions.

Multimodal visualization is typically applied in the scientific visualization domain. The integration of information visualization and scientific visualization remains a challenge. In brain connectomics, connectome network’s connectivity data are usually visualized as weighted graphs. Graph visualization has been extensively studied in information visualization. There are some works that focus on visual comparison of different weighted graphs. For example, Alper et al. [15] used superimposed matrix representations to visually compare the difference of two connectome networks. Yang et al. [16] improved and designed a two-step hierarchical strategy and NodeTrix representation to get a better result. For connectomics application, the networks can be either visualized as separate graphs, away from the anatomical context, but connected through interactive interfaces [8–11] or embedded into the brain anatomical context [17–19]. The embedded graphs, however, have their nodes constrained to their anatomical locations and therefore do not need a separate graph layout process as in other graph visualization algorithms. Aside from embedded graphs, there has been little work in integrating more sophisticated information visualization, such as time series data and multi-dimensional attributes, within the context of brain anatomical structures.

Many visualization techniques for time series data have been developed in information visualization, such as time series plot [20], spiral curves [21], and ThemeRiver [22], for non-spatial information and time-variant attributes. Several variations of ThemeRiver styled techniques have been applied in different time series visualization applications, in particular in text visualization [23]. Depicting connectivity dynamics has been mostly done via traditional key-frame-based approach [24, 25] or key frames combined with time series plots [26, 27].

Texture-based visualization techniques have been widely used for vector field data, in particular, flow visualization. Typically, a grayscale texture is smeared in the direction of the vector field by a convolution filter, for example, the line integral convolution (LIC), such that the texture reflects the properties of the vector field [28–30]. Similar techniques have also been applied to tensor fields [31, 32].

As to volume datasets, volume rendering is a classic visualization technique. Both image-space and object-space volume rendering algorithms have been thoroughly studied in the past several decades. The typical image-space algorithm is ray casting, which was first proposed by Levoy [33]. Many improvements in ray casting have since been developed [34–37]. Splatting is the most common object-space approach. It directly projects voxels to the 2D screen to create screen footprints which can be blended to form composite images [38–42]. Hybrid approaches such as shear-wrap algorithm [43] and GPU-based algorithms provide significant speedup for interactive applications [44, 45]. Although iso-surfaces are typically extracted from volume data as polygon meshes [46], ray casting methods can also be applied toward volumetric iso-surfacing [47, 48].

There are a few freely available toolkits for visualizing human brain data. MRIcron (http://people.cas.sc.edu/rorden/mricron/) is a convenient tool to view 2D slices of MRI data. TrackVis (http://trackvis.org/) can visualize DTI fiber tracts with MRI data as background in 3D view. FSLView (https://fsl.fmrib.ox.ac.uk/fsl/fslwiki/FslView) is a submodule of the FSL library which can do 2D/3D rendering of MRI and functional MRI (fMRI) data. Braviz (http://diego0020.github.io/braviz/) is a visual analytics tool supporting the visualization of MRI, fMRI, and DTI data including fiber tracts. While these tools are excellent at visualizing individual modalities separately, they typically do not emphasize on the functionality of an integrated visualization of all kinds of brain data within the context of the same anatomical background.

## Brain imaging data and connectome construction

We first describe the MRI and DTI data used in this study, then present our methods for constructing connectome networks from the MRI and DTI data, and finally discuss the resting-state functional MRI (fMRI) data used in our time series visualization study.

### MRI and DTI data from the ADNI cohort

The MRI and DTI data used in the preparation of this article were obtained from the Alzheimer’s Disease Neuroimaging Initiative (ADNI) database (adni.loni.usc.edu). The ADNI was launched in 2003 as a public–private partnership, led by Principal Investigator Michael W. Weiner, MD. The primary goal of ADNI has been to test whether serial MRI, positron emission tomography (PET), other biological markers, and clinical and neuropsychological assessment can be combined to measure the progression of mild cognitive impairment (MCI) and early Alzheimer’s disease (AD). For up-to-date information, see www.adni-info.org.

We downloaded the baseline 3T MRI (SPGR) and DTI scans together with the corresponding clinical data of 134 ADNI participants, including 30 cognitively normal older adults without complaints (CN), 31 cognitively normal older adults with significant memory concerns (SMC), 15 early MCI (EMCI), 35 late MCI (LMCI), and 23 AD participants. In our multi-class disease classification experiment, we group these subjects into three categories: healthy control (HC, including both CN and SMC participants, *N* = 61), MCI (including both EMCI and LMCI participants, *N* = 50), and AD (*N* = 23).

Using their MRI and DTI data, we constructed a structural connectivity network for each of the above 134 participants. Our processing pipeline is divided into three major steps described below: (1) generation of regions of interest (ROIs), (2) DTI tractography, and (3) connectivity network construction.

1. ROI generation. Anatomical parcellation was performed on the high-resolution T1-weighted anatomical MRI scan. The parcellation is an automated operation on each subject to obtain 68 gyral-based ROIs, with 34 cortical ROIs in each hemisphere, using the FreeSurfer software package (http://freesurfer.net/). The Lausanne parcellation scheme [48] was applied to further subdivide these ROIs into smaller ROIs, so that brain networks at different scales (e.g., $N_{\text{roi}}$ = 83, 129, 234, 463, or 1015 ROIs/nodes) could be constructed. The T1-weighted MRI image was registered to the low resolution b0 image of DTI data using the FLIRT toolbox in FSL, and the warping parameters were applied to the ROIs so that a new set of ROIs in the DTI image space were created. These new ROIs were used for constructing the structural network.
2. DTI tractography. The DTI data were analyzed using FSL. Preprocessing included correction for motion and eddy current effects in DTI images. The processed images were then output to Diffusion Toolkit (http://trackvis.org/) for fiber tracking, using the streamline tractography algorithm called FACT (fiber assignment by continuous tracking). The FACT algorithm initializes tracks from many seed points and propagates these tracks along the vector of the largest principle axis within each voxel until certain termination criteria are met. In our study, stop angle threshold was set to 35 degree, which meant if the angle change between two voxels was greater than 35 degree, the tracking process stopped. A spline filtering was then applied to smooth the tracks.
3. Network Construction. Nodes and edges are defined from the previous results in constructing the weighted, undirected network. The nodes are chosen to be $N_{\text{roi}}$ ROIs obtained from Lausanne parcellation. The weight of the edge between each pair of nodes is defined as the density of the fibers connecting the pair, which is the number of tracks between two ROIs divided by the mean volume of two ROIs [49]. A fiber is considered to connect two ROIs if and only if its end points fall in two ROIs, respectively. The weighted network can be described by a matrix. The rows and columns correspond to the nodes, and the elements of the matrix correspond to the weights.

To demonstrate our visualization scheme for integrative exploration of the time series of resting-state fMRI (rs-fMRI) data with brain anatomy, we employed an additional local (non-ADNI) subject, who was scanned in a Siemens PRISMA 3T scanner (Erlangen Germany). A T1-weighted sagittal MP-RAGE was obtained (TE = 2.98 ms, TR partition = 2300 ms, TI = 900 ms, flip angle = 9°, 128 slices with 1 × 1 × 1 mm voxels). A resting-state session of 10 min was also obtained. Subject was asked to stay still and awake and to keep eyes closed. BOLD acquisition parameters were: TE = 29 ms, TR = 1.25 s, flip angle = 79°, 41 contiguous interleaved 2.5 mm axial slices, with in-plane resolution = 2.5 × 2.5 mm. BOLD time series acquired were then processed according to the following steps (for details see [50]): mode 1000 normalization; z-scoring and detrending; regression of 18 detrended nuisance variables (6 motion regressors [*X Y Z* pitch jaw roll], average gray matter (GM), white matter (WM) and cerebral spinal fluid (CSF) signals, and all their derivatives computed as backwards difference); band-pass filter of 0.009 to 0.08 Hz using a zero-phase second-order Butterworth filter; spatial blurring using a Gaussian filter (FWHM = 2 mm); regression of the first 3 principal components of WM (mask eroded 3 times) and CSF (ventricles only, mask eroded 1 time). The Desikan-Killiany Atlas (68 cortical ROIs, as available in the FreeSurfer software) was registered to the subject. The resulting processed BOLD time series where then averaged for each ROI. Note that the Lausanne parcellation scheme (mentioned above) at the level of $N_{\text{roi}}$ = 83 consists of the above 68 cortical ROIs together with the brain stem (as 1 ROI) and 14 subcortical ROIs. As a result, we will use 68 time series (one for each cortical ROI) in our time series visualization experiments.

## Information visualization: methods and results

In this section, we propose a few information visualization methods. We have implemented and packaged these methods into a software tool named as BECA, standing for Brain Explorer for Connectomic Analysis. A prototype software is available at http://www.iu.edu/~beca/.

### Visualizing structural connectivity networks

**Fig. 1:**
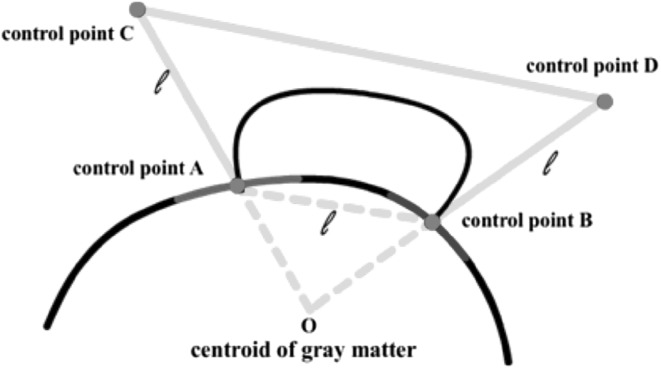
Building a Bezier curve connecting two ROIs

**Fig. 2:**
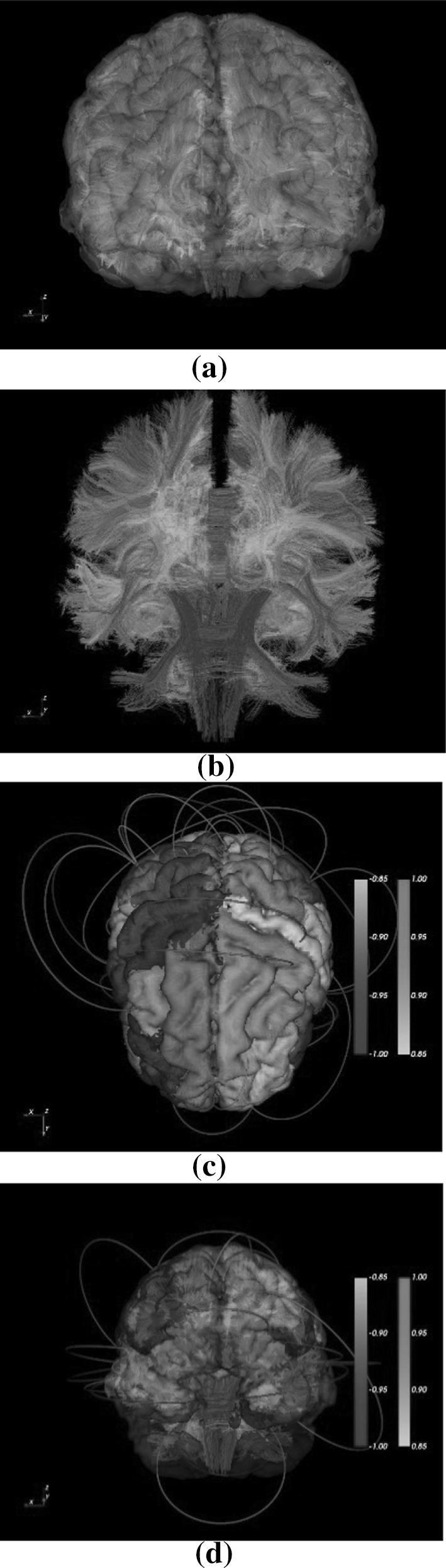
**a** DTI fiber tracts, **b** MRI-ROIs and DTI fibers, **c**, **d** network edges as Bezier curves (thresholded by edge intensity)

3D visualization of a connectivity network within an anatomical structure can provide valuable insight and better understanding of the brain networks and their functions. In a brain network, we render nodes as ROI surfaces, which are generated using an iso-surface extraction algorithm from the MRI voxel sets of the ROIs. Drawing the network edges is, however, more challenging since straight edges will be buried inside the brain structures. We apply the cubic Bezier curves to draw curved edges above the brain structure. The four control points of each edge are defined by the centers of the ROI surfaces and the extension points from the centroid of the brain, as shown in Fig. 1. Figure 2 shows visualization examples of a connectome network, along with the cortical surface, the ROIs, and the DTI fibers.

Brain connectivity networks obtained through the above pipeline can be further taken into complex network analysis. Network measures (e.g., node degree, betweenness, closeness) can be calculated from individuals or average of a population. Different measures may characterize different aspects of the brain connectivity [51]. In order to visualize these network attributes, we propose a surface texture-based approach. The main idea is to take advantage of the available surface area of each ROI and encode the attribute information in a texture image, and then texture-map this image to the ROI surface. Since the surface shape of each ROI (as a triangle mesh) is highly irregular, it becomes difficult to assign texture coordinates for mapping the texture images. We apply a simple projection plane technique. A projection plane of an ROI is defined as the plane with a normal vector that connects the center of the ROI surface and the centroid of the entire brain. The ROI surface can then be projected onto its projection plane, and the reverse projection defines the texture mapping process. Thus, we can define our attribute-encoded texture image on this project plane to depict a visual pattern on the ROI surface. Visually encoding attribute information onto a texture image is an effective way to represent multiple attributes or time series attributes. Below we will demonstrate this idea in two different scenarios: time series data from rs-fMRI and multi-class disease classification.

### Visualizing fMRI data and functional connectivity

As a functional imaging method, rs-fMRI can measure interactions between ROIs when a subject is resting [52]. Resting brain activity is observed through changes in blood flow in the brain which can be measured using fMRI. The resting-state approach is useful to explore the brain’s functional organization and to examine whether it is altered in neurological or psychiatric diseases. Brain activation levels in each ROI represent a time series that can be analyzed to compute correlations between different ROIs. This correlation-based network represents the functional connectivity networks, and analogously to structural connectivity, it may be represented as a square symmetric matrix.

**Fig. 3:**
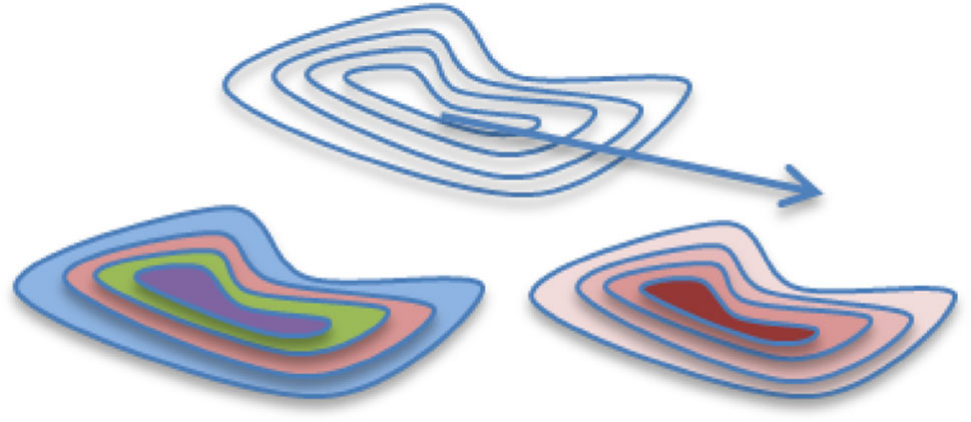
Offset contours with *different colors* or *different shades* of the *same color*

Using the surface texture mapping approach, we need to first encode this time series data on a 2D texture image. We propose an offset contour method to generate patterns of contours based on the boundary of each projected ROI. The offset contours are generated by offsetting the boundary curve toward the interior of the region, creating multiple offset boundary curves, as shown in Fig. 3. There are several offset curve algorithms available in curve/surface modeling. Since in our application, the offset curves do not need to be very accurate, we opt to use a simple image erosion algorithm [53] directly on the 2D image of the map to generate the offset contours.

**Fig. 4:**
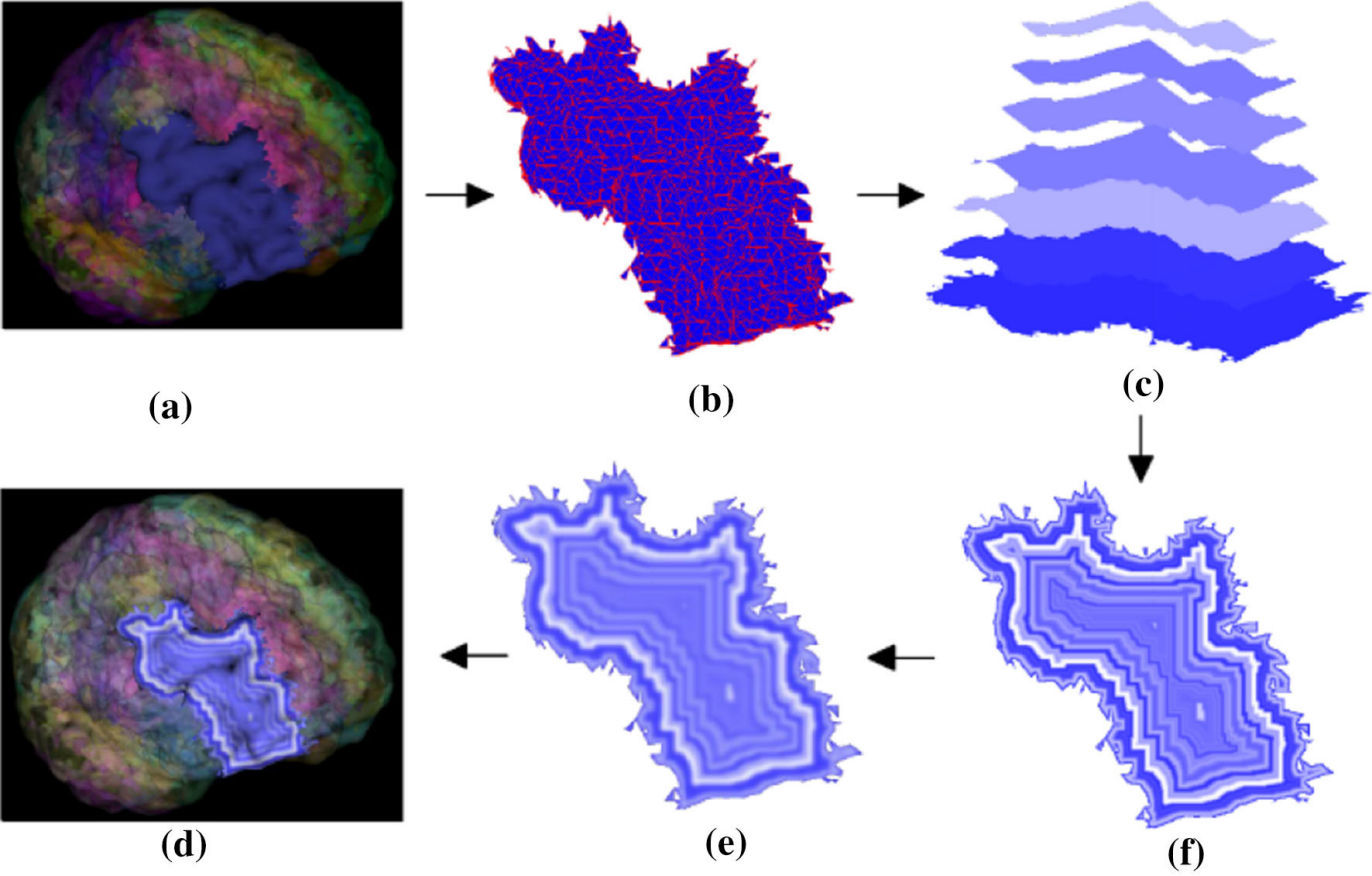
**a** Original ROI, **b** ROI mapping, **c** iterative erosion, **d** overlaying, **e** Gaussian blurring, **f** applying the texture

**Fig. 5:**
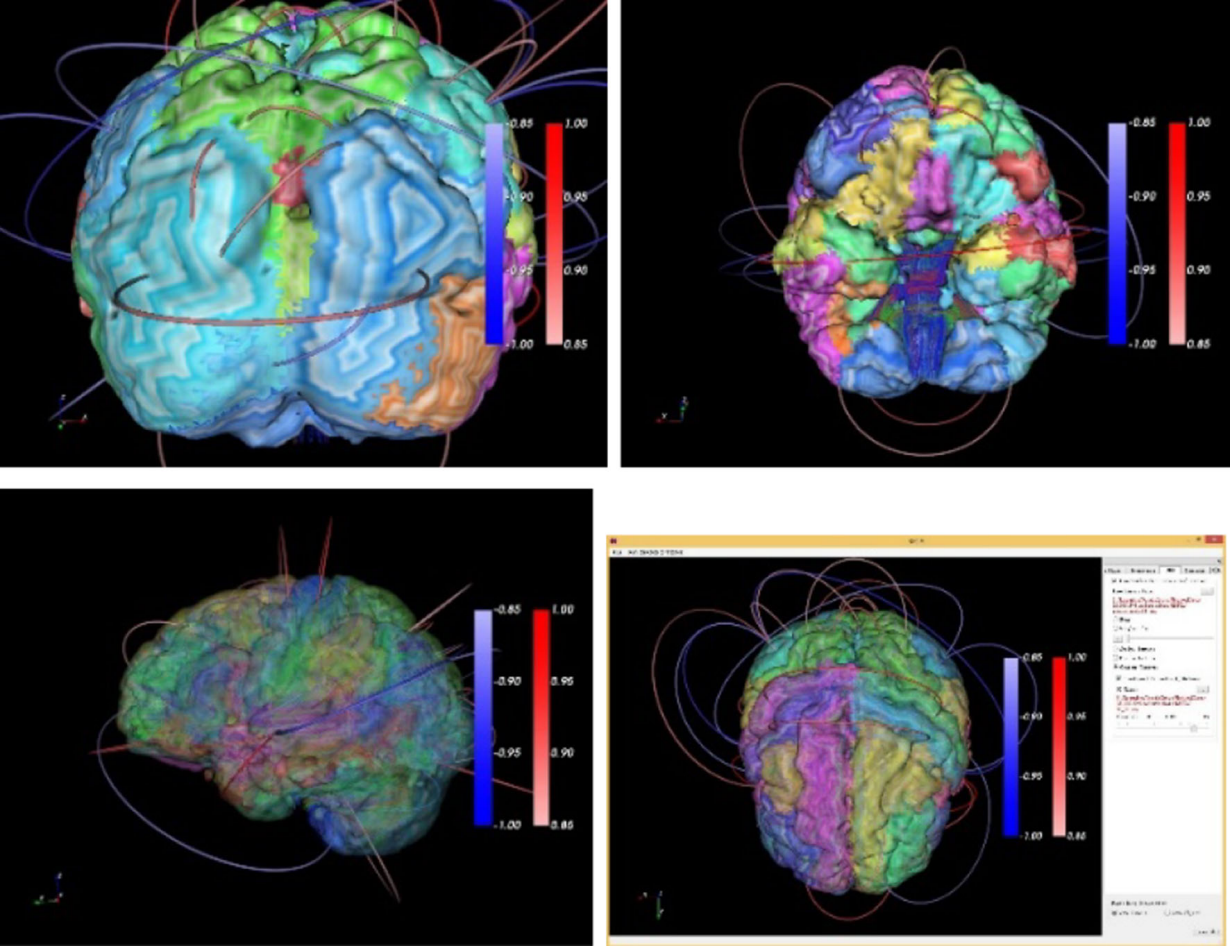
Some examples of a connectome network with time series data. Various transparencies are applied

In time series data visualization, the time dimension can be divided into multiple time intervals and represented by the offset contours. Varying shades of a color hue can be used to represent the attribute changes over time. Figure 4 shows the steps for constructing the contour-based texture. First, we map each ROI onto a projection plane perpendicular to the line connecting the centroid of the brain and the center of this ROI. The algorithm then iteratively erodes the mapped shape and assigns colors according to the activity level of this ROI at each time point. Lastly we overlay the eroded regions to generate a contour-based texture. We also apply a Gaussian filter to smooth the eroded texture image to generate more gradual changes in the activities over time. Figure 5 shows a few examples of the offset contours mapped to the ROIs. The original data have 632 time points, which will be divided evenly across the contours depending on the number of contours that can be fitted into the available pixels within the projected ROI.

### Visualizing discriminative patterns among multiple classes

In this case study, we performed the experiment on the ADNI cohort mentioned before, including 61 HC, 50 MCI, and 23 AD participants. The goal is to generate intuitive visualization to provide cognitively intuitive evidence for discriminating ROIs that can separate subjects in different classes. This can be the first step of a diagnostic biomarker discovery process.

The goal of the visual encoding in this case is to generate a color pattern that can easily distinguish bias toward any of the three classes. To do so, we first assign a distinct color to each class. Various color patterns can be generated using different color blending and distribution methods. In our experiment, a noise pattern is applied with three colors representing the three classes. The same noise pattern approach can also accommodate more colors.

Since color blending is involved in a noise pattern, we choose to use an RYB color model, instead of the RGB model. This is because color mix using RYB model is more intuitive in a way that the mixed colors still carry the proper amount of color hues of the original color components. For example, red and yellow mix to form orange, and blue and red mix to form purple. Thus, RYB model can create color mixtures that more closely resemble the expectations of a viewer. Of course these RYB colors still need to be eventually converted into the RGB values for display. For the conversion between these two color models, we adopt the approach proposed in [54, 55], in which a color cube is used to model the relationship between RYB and RGB values. For each RYB color, its approximated RGB value can be computed by a trilinear interpolation in the RYB color cube.

**Fig. 6:**
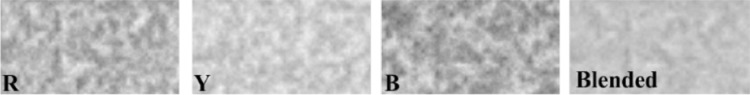
Blending RYB channels with weights 0.5, 0.25, and 0.25

We first construct noise patterns to create a random variation in color intensity, similar to the approach in [54]. Different color hues are used to represent the attributes in different classes of subjects. Any network measurement can be used for color mapping. In our experiment, we use the node degrees averaged across subjects in each class. A turbulence function [56] is used to generate the noise patterns of different frequencies (sizes of the subregions of the noise pattern). An example is shown in Fig. 6; we blend RYB channels with weights 0.5, 0.25, and 0.25, respectively. The blended texture is red-dominated with a little yellow and blue color.

**Fig. 7:**
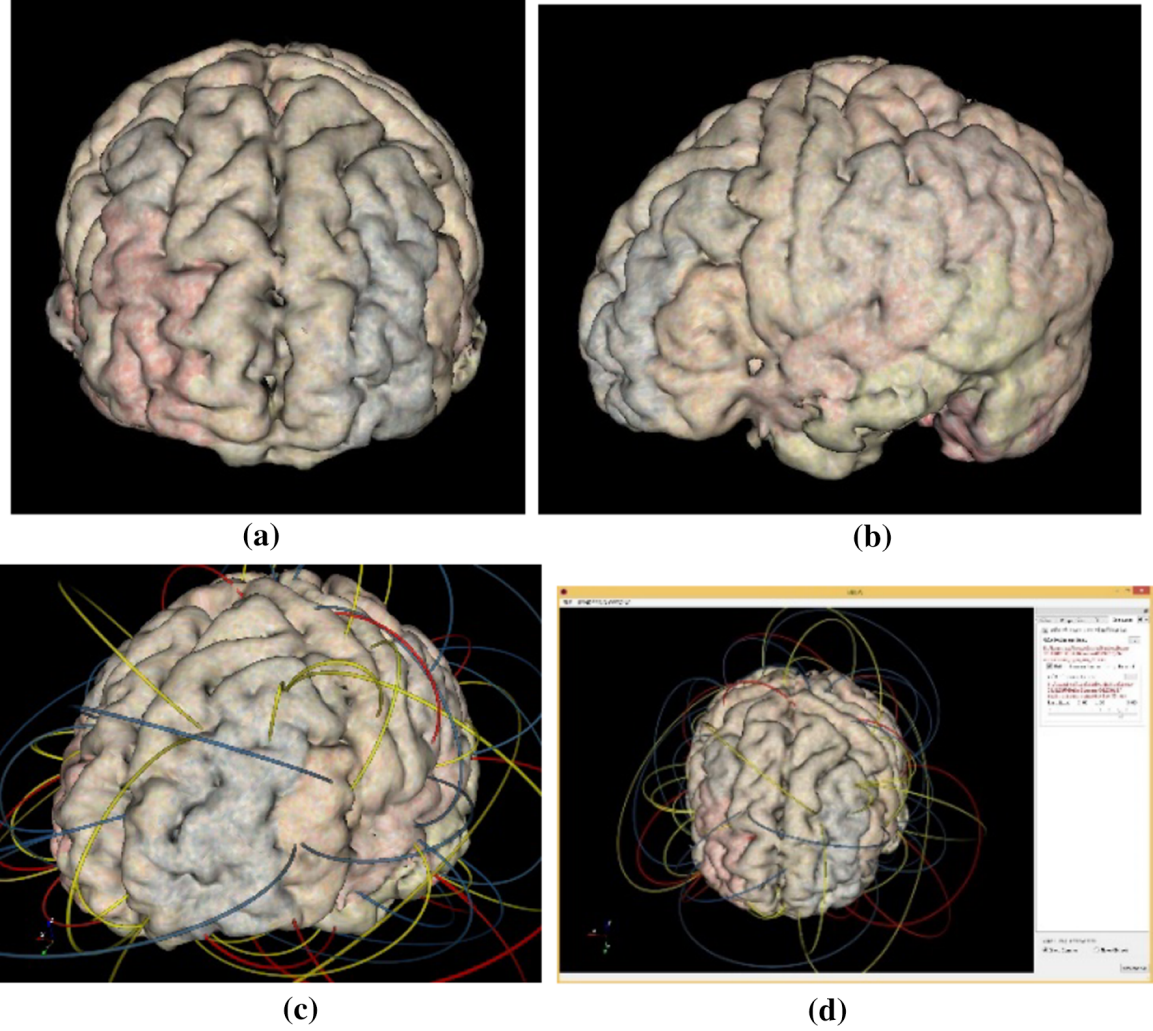
Examples of connectome networks with noise patterns: **a**, **b** ROIs with noise textures; **c**, **d** ROIs with noise textures and color bended edges

Figure 7 shows some examples of the texture mapped views of the three classes: HC (red), MCI (yellow), and AD (blue). The colors of the edges also represent the blended RYB color values, based on the average edge weights in the three classes. From the resulting images, we can identify a specific ROI that exhibits bias toward one or two base colors. This can be a potential indication that this ROI may be a good candidate for further analysis as a potential imaging phenotypic biomarker.

## Spherical volume rendering (SVR)

In previous sections, we mapped attributes onto the ROI surface. However, each rendering shows only one perspective, and subcortical structures remain unseen. Therefore, it does not provide an overall view of the complete structure. In this section, we develop a spherical volume rendering algorithm that provides a single 2D map of the entire brain volume to provide better support for global visual evaluation and feature selection for analysis purpose.

Traditional volume rendering projects voxels to a 2D screen defined in a specific viewing direction. Each new viewing direction will require a new rendering. Therefore, users need to continuously rotate and transform the volumetric object to generate different views, but never have the complete view in one image. Spherical volume rendering employs a spherical camera with a spherical screen. Thus, the projection process only happens once, providing a complete image from all angles.

### Spherical ray casting

A spherical ray casting approach is taken to produce a rendering image on a spherical surface. A map projection will then be applied to construct a planar image (volume map). The algorithm includes three main steps:

1. Define a globe as a sphere containing the volume. The center and radius of the sphere may be predefined or adjusted interactively.
2. Apply spherical ray casting to produce an image on the globe’s spherical surface (ray casting algorithm).
3. Apply a map projection to unwrap the spherical surface onto a planar image (similar to the world map).

**Fig. 8:**
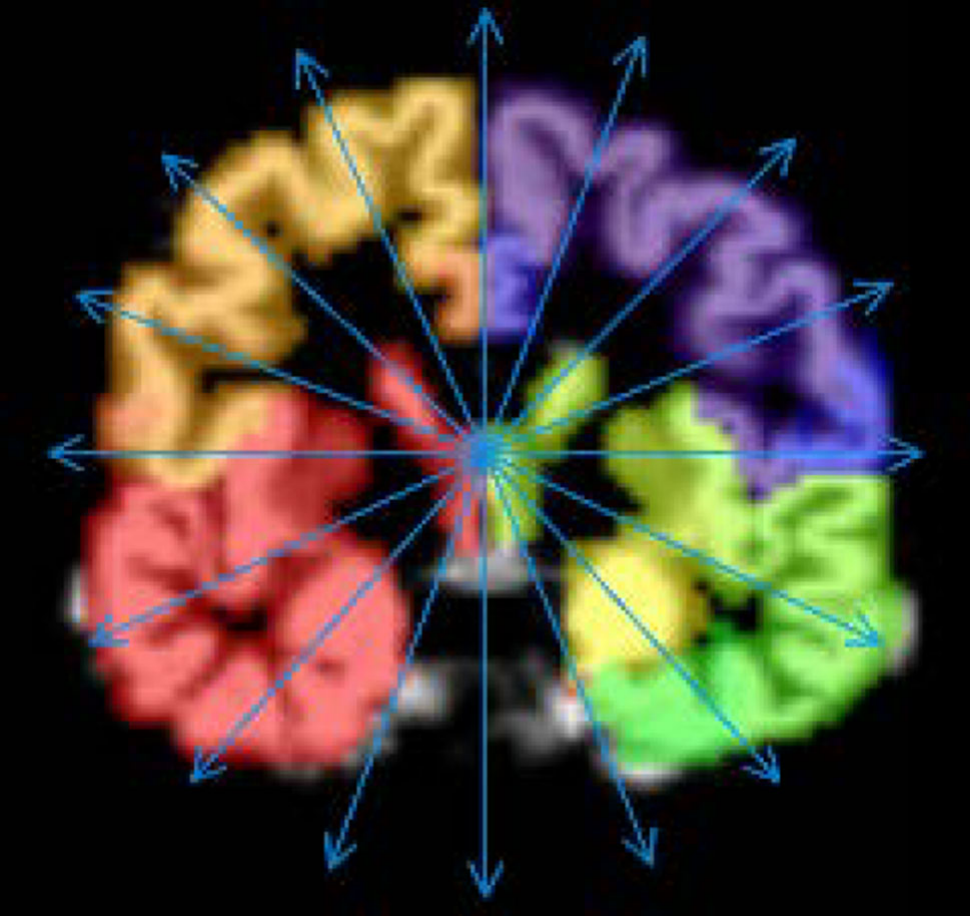
Ray casting toward the center of the brain (sliced)

Rays are casted toward the center of the global from each latitude–longitude grid point on the sphere surface. In brain applications, the center of the global needs to be carefully defined so that the resulting image preserves proper symmetry, as shown in Fig. 8.

Along each ray, the sampling, shading, and blending process is very similar to the regular ray casting algorithm [33, 36]. The image produced by this ray casting process on the spherical surface will be mapped to a planar image using a map projection transformation, which projects each latitude–longitude grid point on the spherical surface into a location on a planar image. There are many types of map projections, each preserving some properties while tolerating some distortions. For our application, we choose to use Hammer–Aitoff Projection, which preserves areas but not angles. Details of this map projection can be found in [57].

### Layered rendering

Volume rendering often cannot clearly show the deep interior structures. One remedy is to use layered rendering. When objects within the volume are labeled (e.g., segmented brain regions), we can first sort the objects in the spherical viewing direction (i.e., along the radius of the sphere) and then render one layer at a time.

**Fig. 9:**
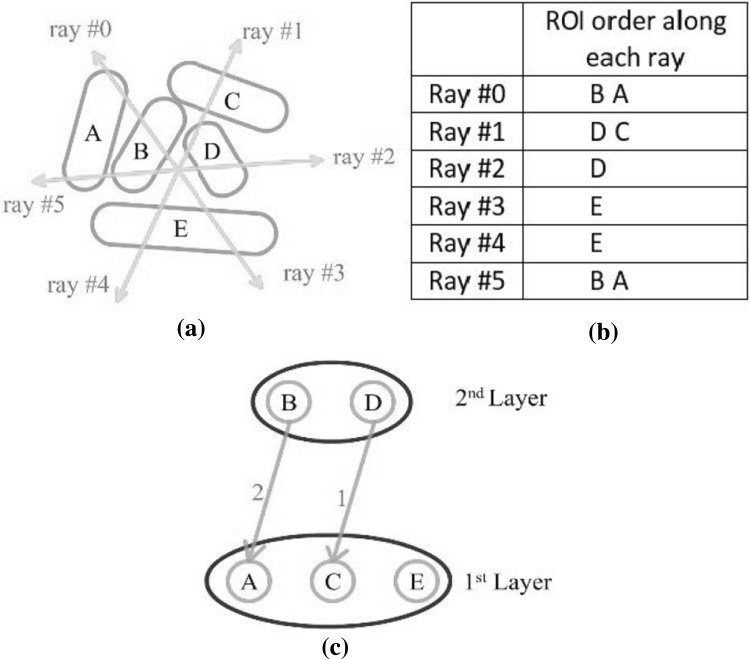
An example of layer sorting for regions of interest (ROIs): **a** 6 Rays and 5 ROIs, **b** occlusion information of each ray, **c** occlusion graph and layers

The spherical viewing order can usually be established by the ray casting process itself as the rays travel through the first layer of objects first, and then the second layer, etc. If we record the orders in which rays travel through these objects, we can construct a directed graph based on their occlusion relationships, as shown in Fig. 9. Applying a topological sorting on the nodes of this graph will lead to the correct viewing order.

Since the shapes of these labeled objects may not be regular or even convex, the occlusion orders recorded by the rays may contradict each other (e.g., cyclic occlusions). Our solution is to define the weight of each directed edge as the number of rays that recorded this occlusion relationship. During the topologic sorting, the node with minimum combined incoming edge weight will be picked each time. This way, incorrect occlusion relationship will be kept to the minimum.

### Brain map by SVR

Using a spherical volume rendering algorithm, we can generate a 2D brain map that contains all the ROIs in one image. This allows the users to view clearly relationships between different ROIs and the global distributions of network attributes and measurements for feature selection and comparison.

**Fig. 10:**
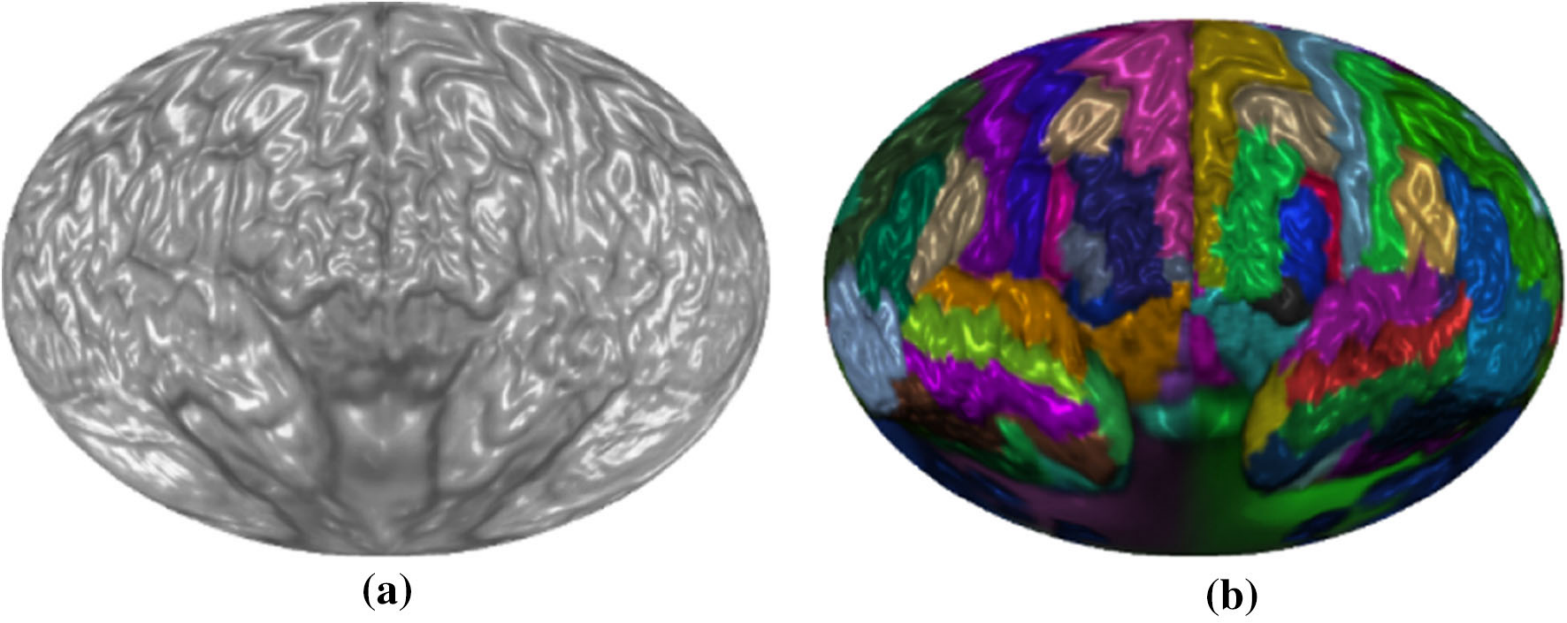
A brain map: **a** without ROI labels, **b** with ROI labels

Figure 10a shows a brain map generated by SVR without any ROI labeling. Figure 10b shows the same brain map with color coded ROI labels.

**Fig. 11:**
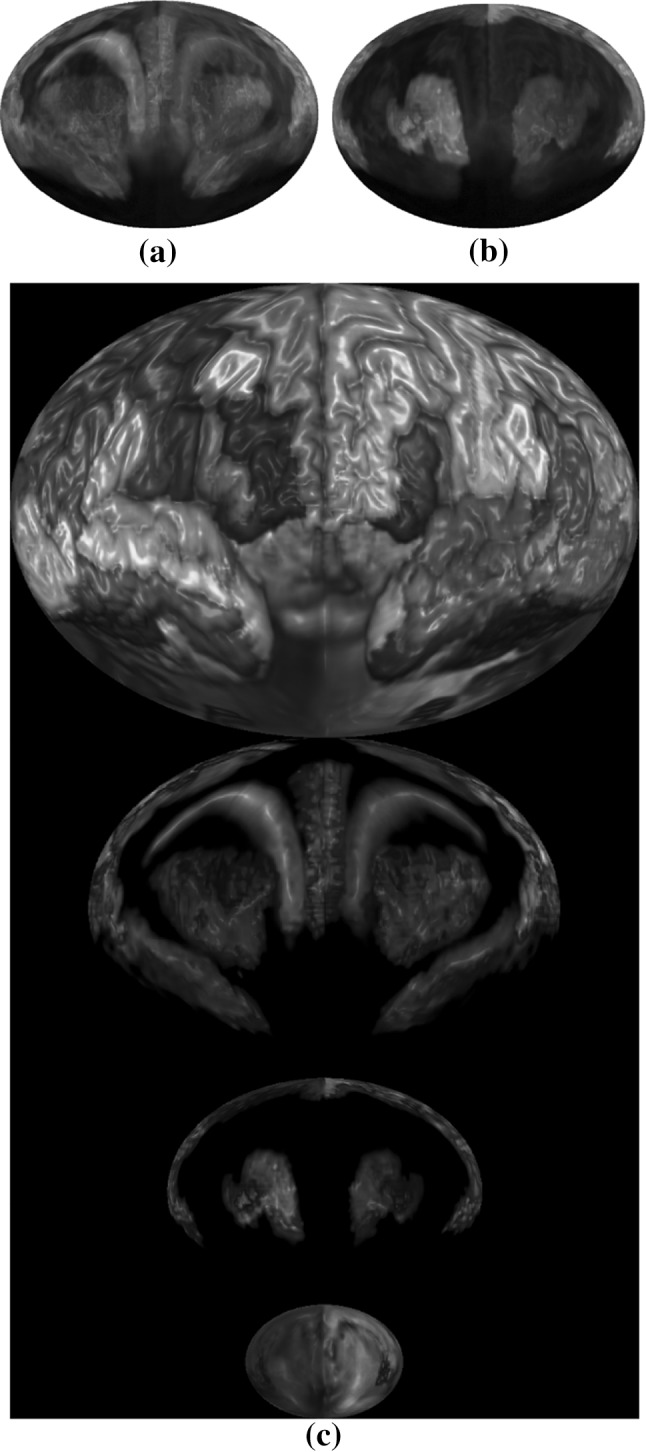
Layers of a brain map: **a** second layer, **b** third layer, **c** all layers stacked

Layered rendering was also applied to brain ROIs. With opacity at 1, Fig. 10 shows the first layer of the ROIs. Figure 11 shows all the layers. Different scaling factors are applied to the layers to adjust their relative sizes. This is necessary because the spherical ray casting will create enlarged internal ROIs and just like perspective projection will make closer objects larger, except that in this case the order is reversed.

In the following two subsections, we demonstrate two approaches to overlay additional information on top of the brain map: (1) encoding attribute information onto a texture image and then mapping the texture to the ROI surface; (2) drawing network edges directly over the brain map. Below, we apply the first approach to an application of visualizing discriminative patterns among multiple classes. In addition, we combine both approaches to visualize fMRI data and the corresponding functional connectivity network.

### Visualizing fMRI data and discriminative pattern

**Fig. 12:**
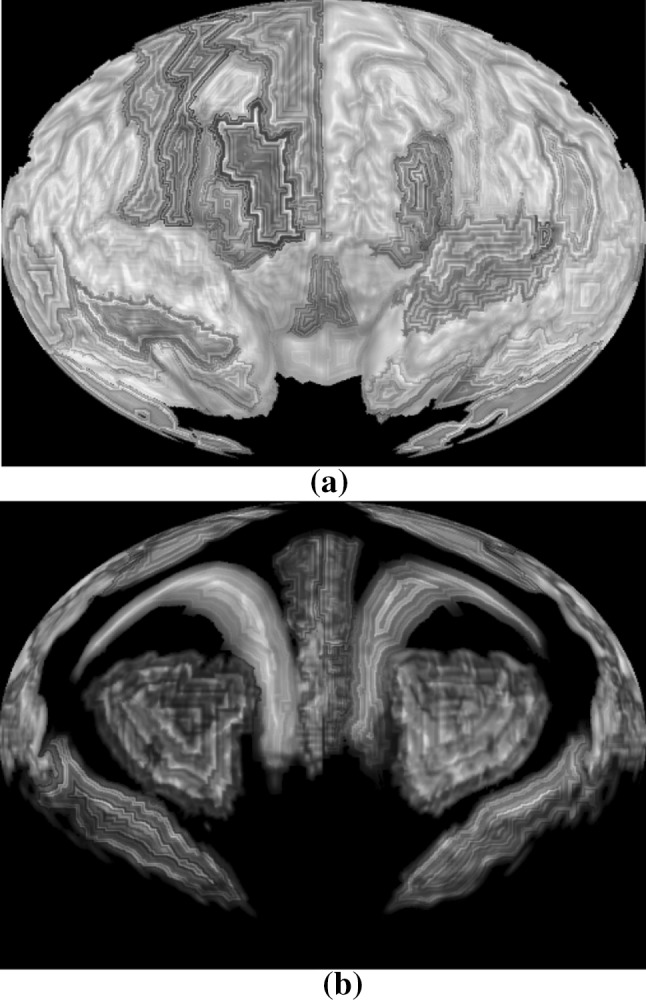
Textured brain map for fMRI data: **a** first layer textures, **b** second layer texture

**Fig. 13:**
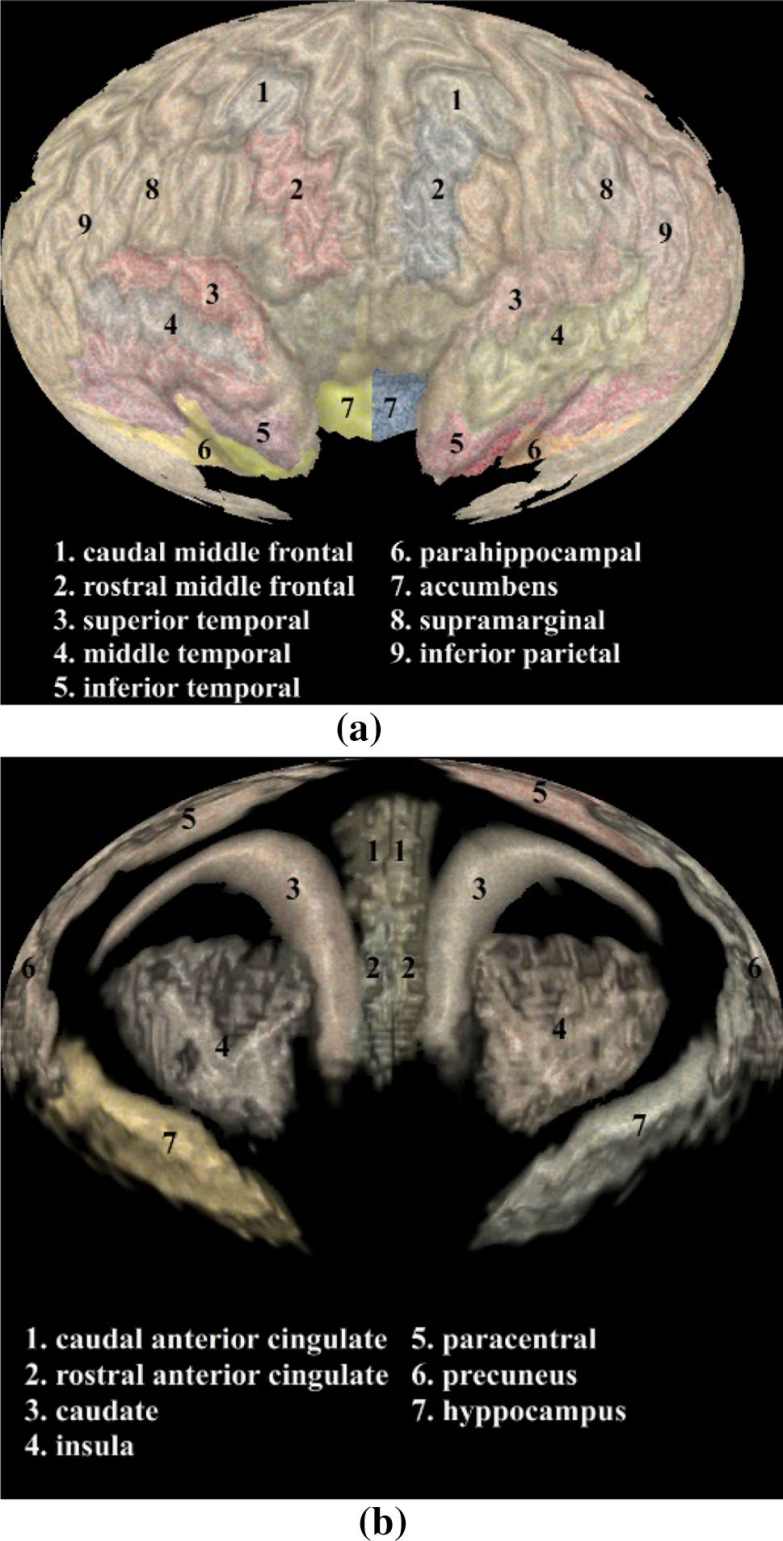
Textured brain map for disease classification: **a** first layer textures, **b** second layer textures. A noise pattern is applied with three colors representing the three categories (i.e., *red* for HC, *yellow* for MCI, and *blue* for AD). (Color figure online)

**Fig. 14:**
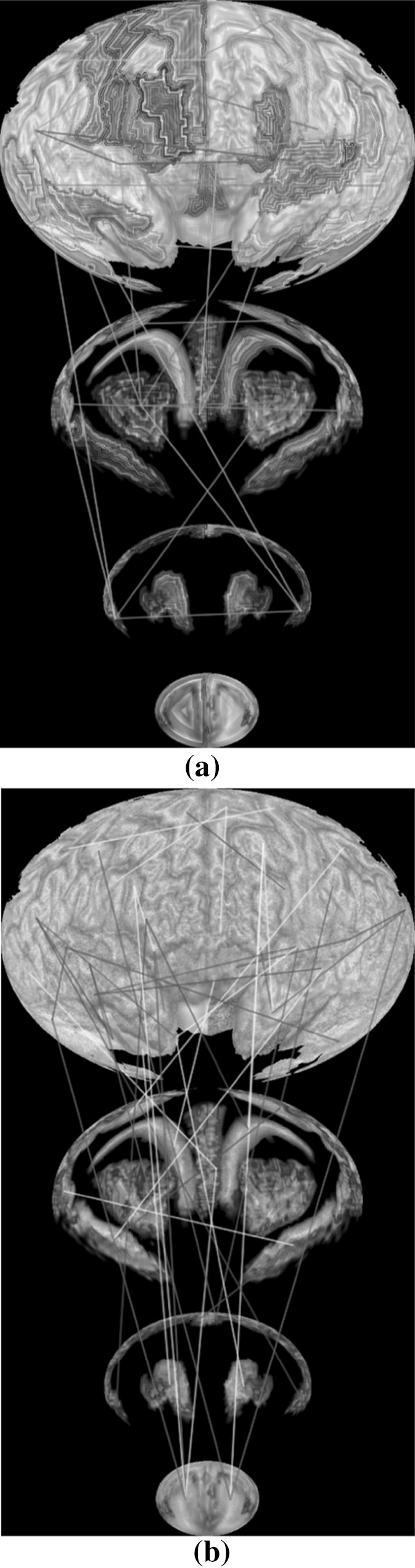
**a** Network edges over multiple layers for time series textures, **b** network edges over multiple layers for multi-disease textures

Figure 12 shows the fMRI textured brain map for the first two layers. Figure 14 shows the network edges across multiple layers for both time series and multi-disease textures (Fig. 13).

### User interface and interaction

**Fig. 15:**
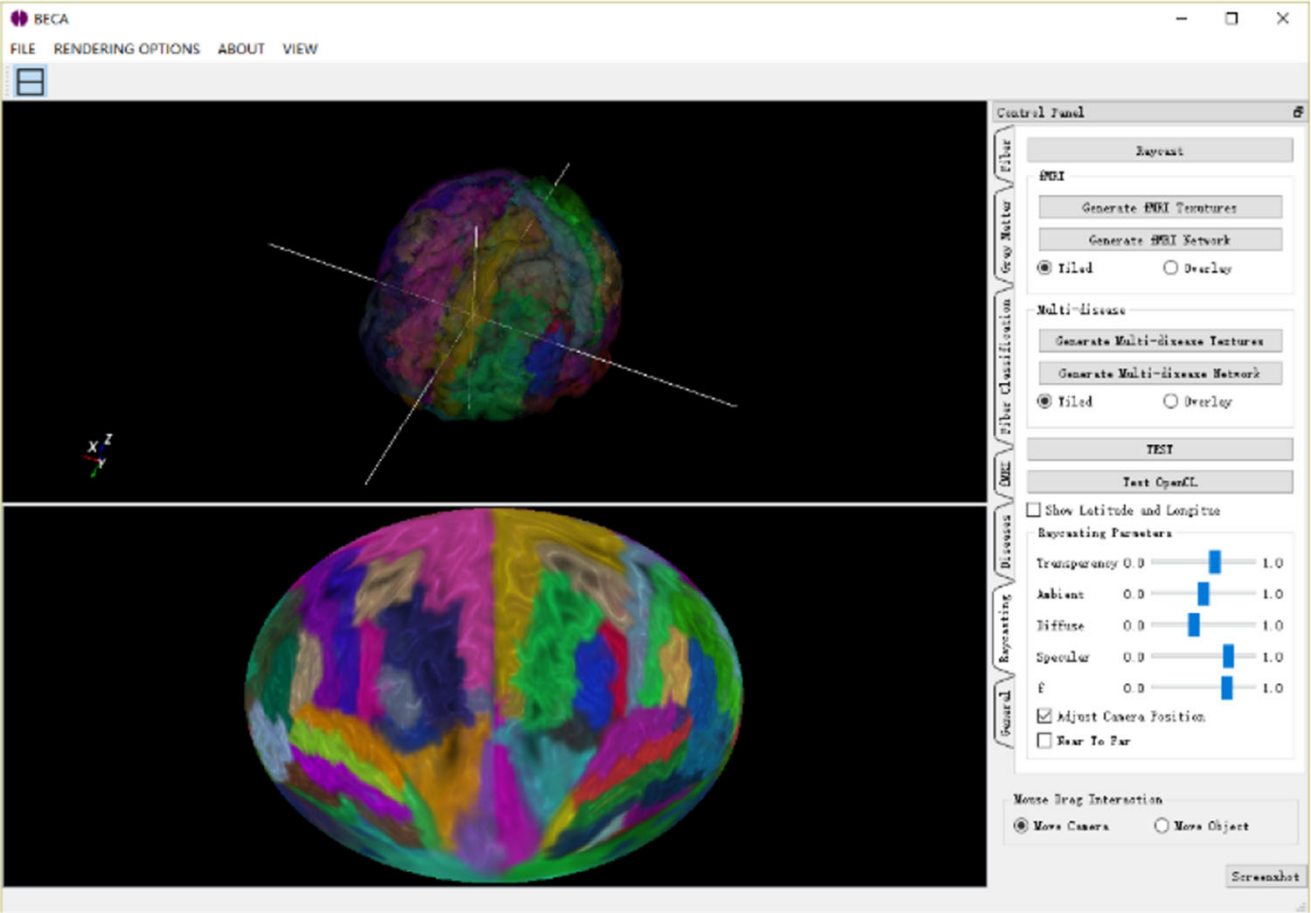
A screenshot of the user interface. When user drag the camera (intersection of the *white lines*) on the *top*, the 2D map on the *bottom* which will be re-rendered in real-time

**Fig. 16:**
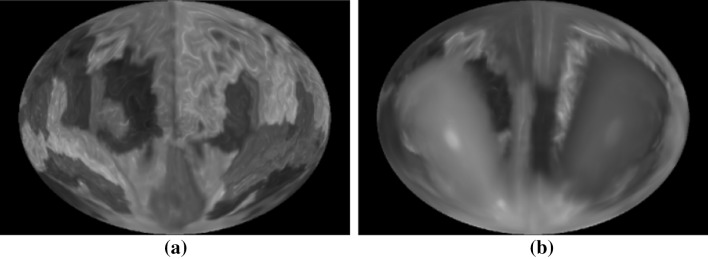
Reverse the direction of ray. **a** Rays travel outwards, **b** rays travel inwards

Compared with traditional volume rendering in the native 3D space, this approach views the brain from its center. On the one hand, this can reduce the volume depth it sees through. On the other hand, it renders ROIs in a polar fashion and arranges ROIs more effectively in a bigger space. With more space available, it is easier to map attributes onto the ROIs and plot the brain networks among ROIs. Compared with traditional 2D image slice view, this approach can render the entire brain using much fewer layers. The user interface (Fig. 15) is flexible enough for users to adjust camera locations and viewing direction. Users can conveniently place the camera into an ideal location to get an optimized view. Users can also easily navigate not only inside but also outside the brain volume to focus on the structures of their interest or view the brain from a unique angle of their interest (Fig. 16).

## Implementation, performance, and evaluation

**Fig. 17:**
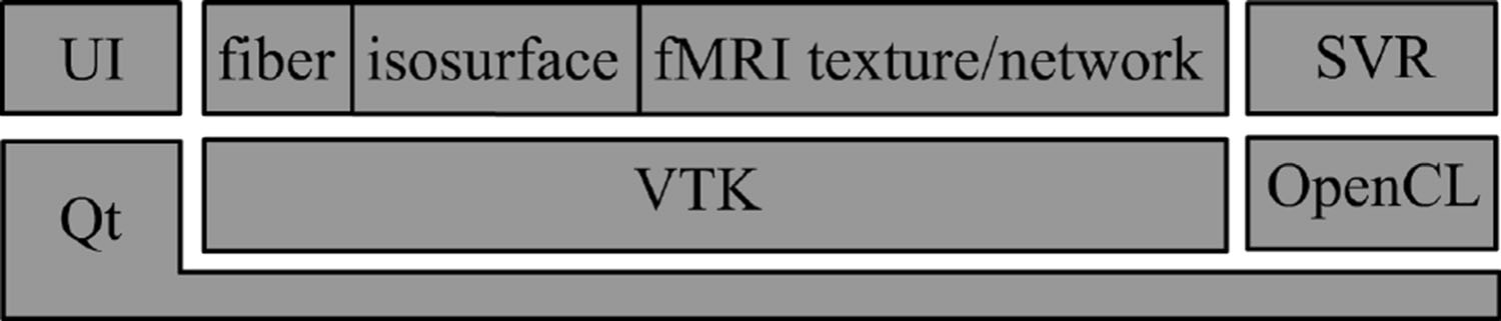
Architecture of the prototype software BECA

An overview of the architecture of the prototype software BECA is illustrated in Fig. 17. The user interface of the prototype software BECA is built with Qt library [58]. The fiber tracts are rendered as polylines by VTK library [59]. The surfaces of brain structures are extracted from MRI scans by vtkMarchingCubes filter and then rendered as vtkPolyData in VTK. fMRI textures are then generated and mapped on the mesh as vtkTexture. The SVR algorithm is implemented on GPU with OpenCL [60] on NVIDIA GeForce GTX 970 graphics card with 4 GB memory. We pass the volume data to kernel function as image3d_t objects in OpenCL in order to make use of the hardware-accelerated bilinear interpolation when sampling along each ray. The normal of each voxel, which is required in Blinn–Phong shading model, is pre-calculated on CPU when the MRI volume is loaded. The normal is also treated as a color image3d objects in OpenCL, which can save lots on time on interpolation. We make each ray one OpenCL work-item in order to render each pixel in parallel. The global work-item size is the size of the viewport. The performance depends on the output image size, which is shown in Table 1. With an 800 × 600 viewport size, the performance is around 29.41 frames per second.

**Table 1:** Frame rates for different output resolutions

Output resolution	Avg. fps
640 × 480	45.45
800 × 600	29.41
1024 × 768	11.76
1600 × 1200	7.04

We have developed tools using Qt framework and VTK to allow user to interact with the 2D map. Users can drag the sphere camera around in the 3D view, and the 2D map will update in real-time. A screenshot of the user interface is shown in Fig. 15. The upper half is the brain in 3D perspective view, while the lower half is the 2D brain map generated by the SVR algorithm. When user moves the position of spherical camera (intersection of the white lines in Fig. 15) in the 3D view, the 2D map will change accordingly. The software enables user to navigate in the 3D brain and builds the visual correspondence between the 3D and 2D representation. We also provide users with a switch to reverse the direction of rays. As shown in Fig. 16a, rays are travels outward and we can see the exterior of the brain. On the contrary, when we reverse the direction of the ray in Fig. 16b, we can see the interior structures of the brain.

We demonstrated our prototype system and the resulting visualization to the domain experts in IU Center for Neuroimaging. The following is a summary of their evaluation comments.

### Evaluation on the visualization of the discriminative pattern

The discriminative pattern shown in Fig. 13 has the promise to guide further detailed analysis for identifying disease-relevant network biomarkers. For example, in a recent Nature Review Neuroscience paper [35], C. Stam reviewed modern network science findings in neurological disorders including Alzheimer’s disease. The most consistent pattern the author identified is the disruption of hub nodes in the temporal, parietal, and frontal regions. In Fig. 13, red regions in superior temporal gyri and inferior temporal gyri indicate that these regions have higher connectivity in HC than MCI and AD. This is in accordance with the findings reported in [35]. In addition, in Fig. 13, the left rostral middle frontal gyrus shows higher connectivity in HC (i.e., red color), while the right rostral middle frontal gyrus shows higher connectivity in AD (i.e., blue color). This also matches the pattern shown in figure 3 of [35], where the hubs at left middle frontal gyrus (MFG) were reported in controls and those at right MFG were reported in AD patients. These encouraging observations demonstrate that our visual discriminative patterns have the potential to guide subsequent analyses.

### Evaluation on the visualization of fMRI data and functional network

It is helpful to see all the fMRI signals on the entire brain in a single 2D image (Fig. 14). Drawing a functional network directly on the flattened spherical volume rendering image (Fig. 14) offers an alternative and effective strategy to present the brain networks. Compared with traditional approach of direct rendering in the 3D brain space, while still maintaining an intuitive anatomically meaningful spatial arrangement, this new approach has more spatial room to work with to render an attractive network visualization on the background of interpretable brain anatomy. The network plot on a multi-layer visualization (Fig. 14) renders the brain connectivity data more clearly and effectively.

### Evaluation on the user interface and interaction

Compared with traditional volume rendering in the native 3D space, this approach views the brain from its center. On the one hand, this can reduce the volume depth it sees through. On the other hand, it renders ROIs in a polar fashion and arranges ROIs more effectively in a bigger space. With more space available, it is easier to map attributes onto the ROIs and plot the brain networks among ROIs. Compared with traditional 2D image slice view, this approach can render the entire brain using much fewer layers (4 in our case) than the number of image slices (e.g., 256 slices in a conformed 1 mm^3^ isotropic brain volume). The user interface (Fig. 15) is flexible enough for users to adjust camera locations and viewing direction. Users can conveniently place the camera into an ideal location to get an optimized view. Users can also easily navigate not only inside but also outside the brain volume to focus on the structures of their interest or view the brain from a unique angle of their interest.

## Conclusions

We have presented an integrated visualization solution for human brain connectome data. Multiple modalities of images are involved including MRI, DTI, and fMRI. Our focus is on the integration of analysis properties of the connectome networks into the anatomical brain structures. We apply a surface texture-based approach to encode network properties and attributes onto the surfaces of the brain structures to establish visual connections and context. Surface texture is an effective approach to integrate information visualization and scientific visualization since scientific data typically have spatial structures containing surface areas, which can be taken advantage of for visual encoding.

In the future, we would like to continue developing the integrated visualization tool for public domain distribution. Currently, a prototype BECA software tool is available at http://www.iu.edu/~beca/, and we will continue improving it. We would also like to study interesting visual analytic topics to compare multiple networks from different network construction procedures, in particular, between structural networks and functional networks.
